# Longitudinal study of pesticide residue levels in human milk from Western Australia during 12 months of lactation: Exposure assessment for infants

**DOI:** 10.1038/srep38355

**Published:** 2016-12-07

**Authors:** Jian Du, Zoya Gridneva, Melvin C. L. Gay, Ching T. Lai, Robert D. Trengove, Peter E. Hartmann, Donna T. Geddes

**Affiliations:** 1 School of Chemistry and Biochemistry, The University of Western Australia, 35 Stirling Hwy, Perth, Western Australia 6009, Australia; 2 Separation Science Laboratory, Research and Development, Murdoch University, 90 South St, Murdoch, Western Australia 6150, Australia; 3 Metabolomics Australia, Murdoch University Node, 90 South St, Murdoch, Western Australia 6150, Australia.

## Abstract

The presence of pesticides in human milk (HM) is of great concern due to the potential health effects for the breastfed infant. To determine the relationships between HM pesticides and infant growth and development, a longitudinal study was conducted. HM samples (n = 99) from 16 mothers were collected at 2, 5, 9 and 12 months of lactation. A validated QuEChERS method and Gas chromatography-tandem mass spectrometry (GC-MS/MS) were used for the analysis of 88 pesticides in HM. Only *p,p’*-DDE, *p,p’*-DDT and *β*-HCH were detected with a mean concentration (±SD) of 52.25 ± 49.88 ng/g fat, 27.67 ± 20.96 ng/g fat and 48.00 ± 22.46 ng/g fat respectively. The concentrations of the detected pesticides decreased significantly throughout the first year of lactation. No significant relationships between HM *p,p’*-DDE and infant growth outcomes: weight, length, head circumference and percentage fat mass were detected. The actual daily intake (ADI) of total DDTs in this cohort was 14–1000 times lower than the threshold reference and significantly lower than the estimated daily intake (EDI). Further, the ADI decreased significantly throughout the first 12 months of lactation.

Persistent organic pollutants (POPs), such as organochlorine pesticides (OCPs), organophosphate pesticides (OPPs), polychlorinated biphenyls (PCBs) and polychlorinated dibenzo-p-dioxins (dioxins), are synthetic chemicals that are present in the environment^1^,^2^. When introduced into the environment, these chemicals can percolate into the soil and ground water, and can be transported over long distances via atmospheric circulation and water movement. These chemicals are absorbed by inhalation, ingestion and dermal contact^3^,^4^. As these xenobiotics enter the body, they bind to transport proteins such as human blood albumin (HBA), globulins and lipoprotein in the plasma^5^. The more lipophilic chemicals, such as OCPs, are then redistributed and accumulated in tissue compartments with high fat content, such as adipose tissue, the liver, kidneys, brain and breasts^3^. Whereas the more hydrophilic xenobiotics, such as carbamate, are more easily metabolized in the liver and then excreted^6^. In lactating women, these xenobiotics can be transferred from the blood to milk together with other necessary nutrients, precursors for the production of human milk (HM) components^7^,^8^. However, the mechanisms underlying the transfer of these chemicals in HM are not yet known. This paper will focus on the persistent organic pollutants in HM with respect to pesticides.

The presence of pesticides in HM is of great concern due to the potential health effects for the breastfed infant, as many of these pesticides are known to interfere with the function of normal endocrine systems^2^. Exposure to these xenobiotics has been associated with a wide range of adverse effects, such as delayed neurodevelopment, poor cognitive performance and growth retardation during early childhood^9^,^10^,^11^. High prenatal exposure to OPPs and its metabolites has been associated with attention deficit/hyperactivity disorder in children at 5 years of age^12^. Similarly, a longitudinal study of 329 children through to 7 years of age confirmed that high maternal OPPs concentrations were associated with poor intellectual development and cognitive performance^13^. In terms of infant growth or size, high levels of chlorpyrifos in maternal prenatal plasma has been associated with smaller infant birth length and weight^14^ and high concentrations of DDE in maternal prenatal urine with smaller infant birth weight and head circumference^15^.

With respect to recent study postnatal exposure to pesticides, Liu (2016) reported an association between smaller newborn head circumference and the metabolites of OPPs, such as dialkylphosphate (DAP) and diethyl phosphate (DEP) which was more pronounced in the male infant. Follow-up assessment of these infants at 2 years of age showed that prenatal exposure of the fetus to OPPs was associated with delayed adaptive skills whilst postnatal exposure to OPPs were associated with delayed social and motor development skills^16^. Caution must be taken in interpretation of these studies as it is difficult to differentiate between prenatal and postnatal exposure to these pesticides. Further unraveling postnatal infant exposure due to HM or other sources such as food and the environment is even more difficult due to lack of measurement of both volume of HM consumed as well as reliable dietary records. Epidemiological studies of pesticides in HM in relation to postnatal infant growth outcomes are non-existent therefore we lack understanding of the effects of the pesticides in HM on the infant growth and development despite the infant in being more vulnerable to the potential effects due to their immature biological systems and the high levels of enzymes required to detoxify these pesticides^17^.

Pesticides have been extensively used in Western Australia (WA) in the past to protect agricultural products, buildings and households against insects and pests^18^, and high concentrations of pesticides, such as DDT and its metabolites, HCB and dieldrin, have been detected in Western Australian (WA) women’s milk previously^19^,^20^,^21^. The most recent study was conducted in 2002/03^22^, however this result was based on a single pooled milk, making it a conservative estimate for the individual and not allowing for investigation of factors that might influence on HM POPs. Since then little information about these pesticides in women’s milk in WA has been reported.

The aims of this study were to describe the current pesticide concentrations in HM and changes during the first year of lactation in a longitudinal cohort. Associations between pesticide concentrations between infant growth outcomes were investigated along with maternal characteristics. The daily intake of the pesticides was calculated rather than estimated and tracked throughout the first year of lactation.

## Results

### Participants

The demographics and characteristics of the study participants are presented in Table 1. No significant differences were observed in maternal BMI (*P *> 0.96), maternal fat mass (*P *> 0.46) and infant fat mass measured by ultrasound skinfolds (*P *> 0.87) and bioimpedance spectroscopy (BIS; *P *> 0.24) throughout the first year of lactation. Whereas significant differences (*P* < 0.01) were observed in infants’ weight, body length and head circumference throughout the first year of life.

### HM fat content and pesticide concentrations

**Figure 1:**
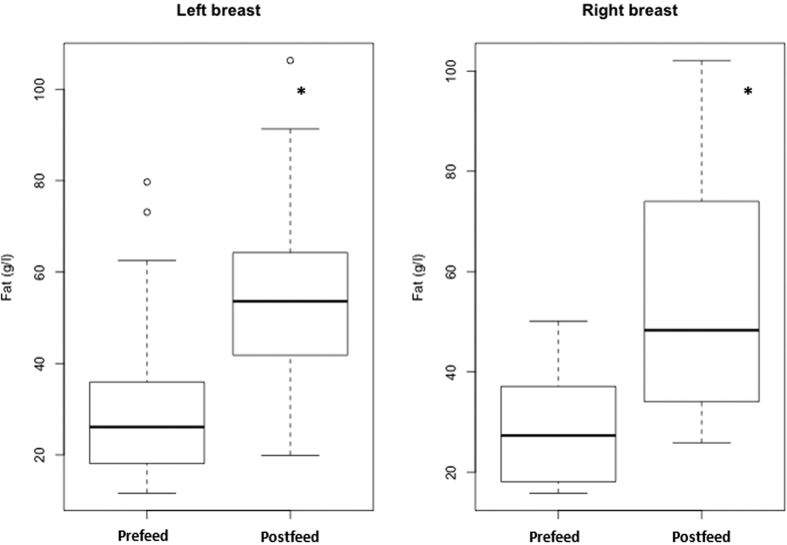
Distribution of fat content in pre- and postfeed milk collected from each breast from 2 to 12 months (n = 99 samples from 16 mothers). The fat contents are shown by box plots illustrating range (error bars), quartiles (box), median (indicated by bold line) and outliers (o). *Indicates significant difference (*P* < 0.05).

The mean fat content of all HM samples (postfeed: n = 39; prefeed: n = 60) was 38.9 ± 21.6 g/L (11.6 to 106.3 g/L), Fat content of postfeed milk (left: 55.1 ± 21.4 g/L; right: 54.9 ± 24.0 g/L) was significantly higher (*P* < 0.01) than prefeed milk (left: 28.8 ± 15.3 g/L; right: 27.6 ± 10.0 g/L) (Fig. 1).

**Figure 2:**
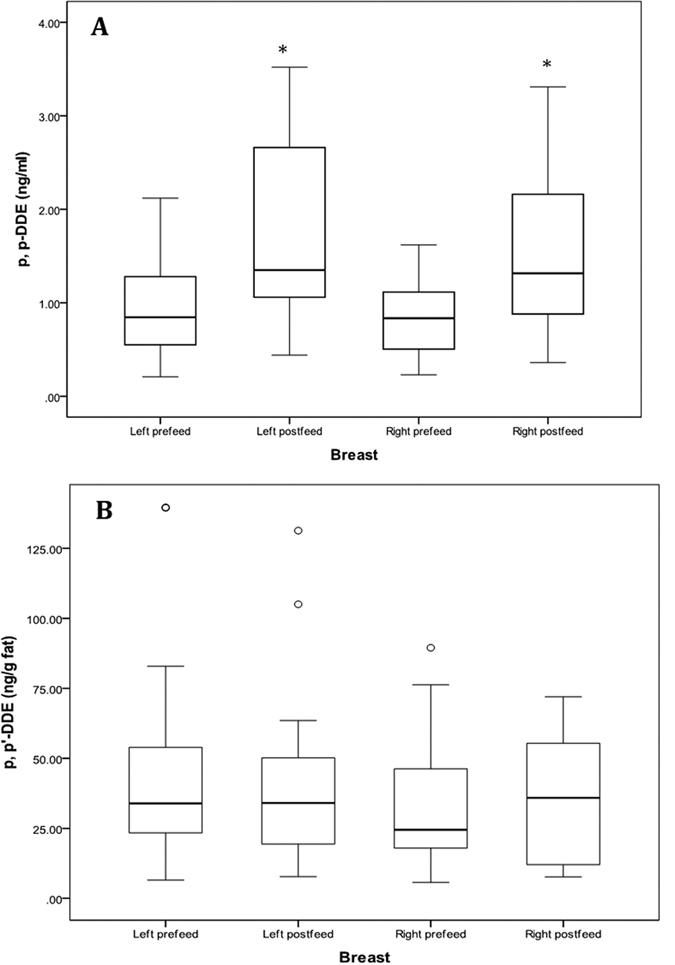
Distribution of *p,p’*-DDE in pre- and postfeed milk collected from each breast before (**A**) and after (**B**) normalized to fat content of HM. Values of *p,p’*-DDE are shown by box plots illustrating range (error bars), quartiles (box), median (indicated by bold line) and outliers (o). *Indicates significant difference (*P* < 0.05).

Only 3 of the 88 pesticides (3.4%), *p,p’*-DDE, *p,p’*-DDT and *β*-HCH, were detected (Table 2). Other pesticides, such as OPPs, fungicides, carbamates and pyrethroids, were not detected. The most frequently detected and abundant pesticide in HM was *p,p’*-DDE; detected in 83 of the 99 samples (83%). The mean concentration of *p,p’*-DDE was 1.56 ± 1.22 ng/mL (range: 0.21–6.21 ng/mL) and when normalized to the HM fat content was 52.25 ± 49.88 ng/g fat (range: 5.67–278.48 ng/g fat). The level of *p,p’*-DDE was significantly higher in the postfeed milk (left: 2.12 ± 1.36 ng/mL; right: 1.57 ± 0.95 ng/mL) compared to the prefeed milk (left: 1.44 ± 1.32 ng/mL; right: 1.08 ± 0.78 ng/mL) (*P* < 0.01; Fig. 2A). After the normalization of *p,p’*-DDE to HM fat content there were no significant differences between the pre- and postfeed *p,p’*-DDE concentrations (left: *P* = 0.24; right: *P* = 0.37; Fig. 2B).

*p,p’*-DDT was detected in the milk of 2 mothers with a mean concentration of 27.67 ± 20.96 ng/g fat (range: 6.08–69.55 ng/g fat) (Table 2). The insecticide, *β*-HCH, was detected in 1 mother, with a mean concentration of 48.00 ± 22.46 ng/g fat (range: 14.95–65.08 ng/g fat) (Table 2).

### Changes in pesticide concentrations during the first year of lactation

**Figure 3:**
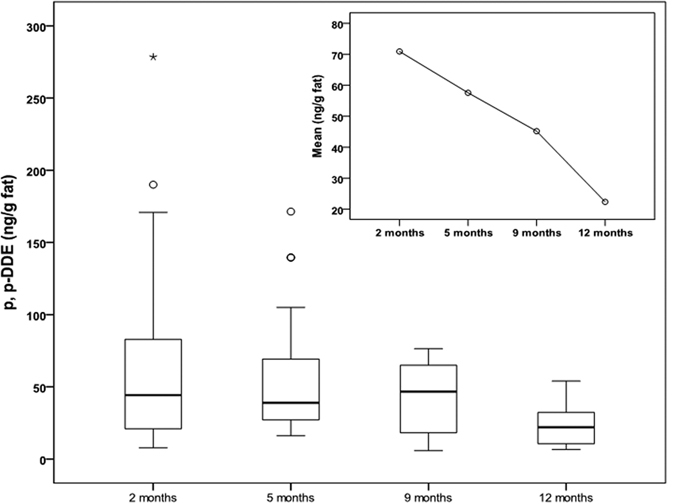
The average concentrations of *p,p’*-DDE (ng/g fat) in HM collected during lactation period from 2 to 12 months. Values of *p,p’*-DDE are shown by box plots illustrating range (error bars), quartiles (box), median (indicated by bold line) and outliers (o). Insert is the mean plot of *p,p’*-DDE levels from this cohort. Significant difference (*P* < 0.05) is observed between 2 and 12 months.

The overall HM *p, p’*-DDE concentrations declined from 70.90 ± 70.58 ng/g fat at 2 months to 57.54 ± 47.32 ng/g fat at 5 months, 45.16 ± 31.71 ng/g fat at 9 months and to 22.35 ± 13.96 ng/g fat at 12 months. There was an overall 68% decrease of *p,p’*-DDE concentrations over the first 12 months of lactation (Fig. 3). A significant difference (*P* = 0.03) was observed in *p,p’*-DDE concentrations between 2 and 12 months only. Similarly, the concentrations of *p,p’*-DDT and *β*-HCH in this study also decreased by 45% and 73% respectively over the lactation period of 2–12 months.

### Pesticide concentrations, maternal and infant characteristics

There were no significant relationships between HM *p,p’*-DDE concentrations and maternal age (*P* = 0.06), parity (*P* = 0.65), maternal body mass index (BMI) (*P* = 0.27) and percentage fat mass (*P* = 0.08). However, mothers of male infants had significantly higher (P = 0.03) concentrations of *p,p’*-DDE in their milk (55.18 ± 45.41 ng/g fat) compared to mothers of female infants (29.34 ± 18.90 ng/g fat) (Supplementary Fig. S1).

Significant increases in infant weight (*P* < 0.01), length (*P* < 0.01) and head circumference (*P* < 0.01) were observed throughout the first year of life (Supplementary Fig. S2). No significant associations were observed between *p,p’*-DDE and the infant growth outcomes; weight (*P* = 0.40), length (*P* = 0.13), head circumference (*P* = 0.07) and percentage fat mass (ultrasound skinfolds: *P* = 0.34; BIS: *P* = 0.11).

### Infant exposure to HM DDT

**Figure 4:**
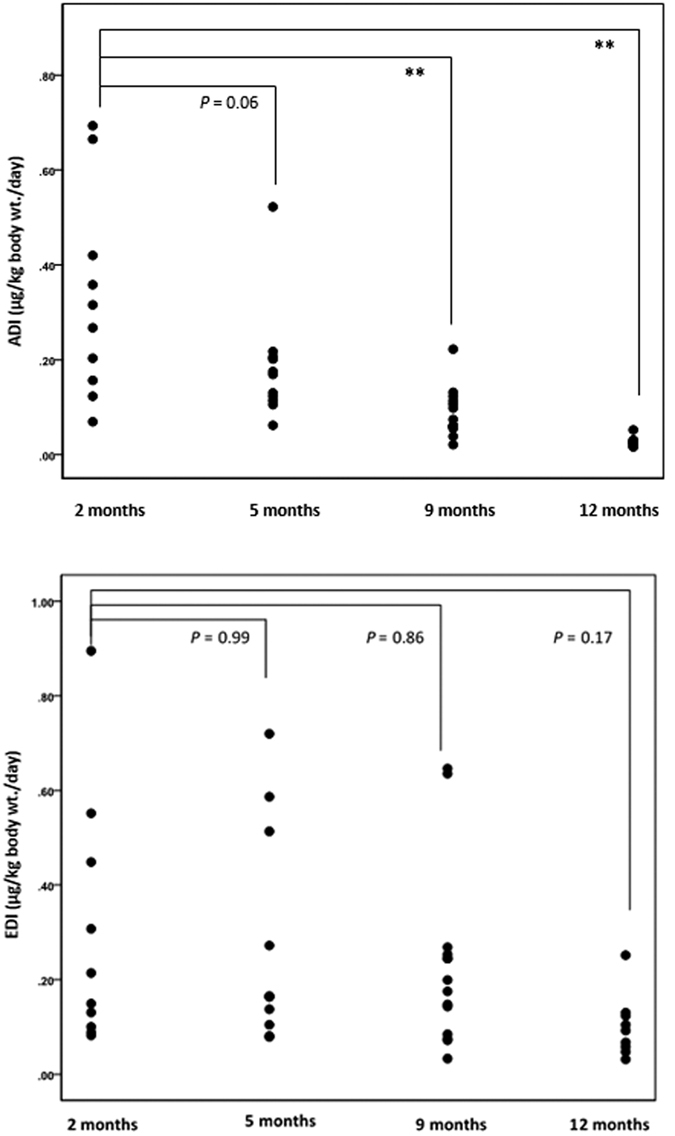
Actual daily intake (ADI) and estimated daily intake (EDI) of DDTs by individual infants throughout the first year of lactation in WA. **Indicates significant difference (*P* < 0.01).

The average calculated actual daily intake (ADI) and estimated daily intake (EDI) of DDTs (sum of DDT and its metabolites, DDE and DDD) throughout the first year of lactation were 0.16 μg/kg body wt./day and 0.23 μg/kg body wt./day, respectively (Table 3). The ADI of DDTs by the infants decreased significantly during the first year of lactation from 0.33 μg/kg body wt./day at 2 months to 0.03 μg/kg body wt./day at 12 month**s** (*P* < 0.01; Fig. 4), which is in conjunction with the infants’ rapid development and also the significant decrease of the maternal bioburden (*P* = 0.03; Fig. 3). No significant difference was observed in the EDI of DDTs by the infants over the 12-month period. The average EDI of DDT at 2 and 5 months (0.27–0.30 μg/kg body wt./day) is comparable to that observed in ADI and drastically overestimates at 9 and 12 months (0.10–0.23 μg/kg body wt./day; *P* < 0.01; Table 3).

## Discussion

This is the first longitudinal study to examine pesticide concentrations in HM and infant growth parameters over the first 12 months of lactation. Only *p,p’*-DDE, *p,p’*-DDT and β*-*HCH in HM were detected out of the 88 pesticides tested with a sensitive and validated QuEChERS (“Quick, Easy, Cheap, Effective, Rugged and Safe”) method. This microscale extraction method is based on the liquid-liquid partitioning of acetonitrile and dispersive solid-phase extraction (dSPE), such as MgSO_4_, sodium acetate, primary secondary amine (PSA), and the pesticides are partitioned into the acetonitrile layer^23^. After normalization of the concentrations to the fat content of HM, the levels of pesticides decreased substantially throughout the first year of lactation and *p,p’*-DDE levels were not related to measures of infant growth such as infant weight, length, head circumference and adiposity.

The HM samples in this cohort were analyzed for pesticides with an optimized QuEChERS methodology^24^. This method had excellent recoveries (70–120%) and detection limits (0.2–2.0 ng/mL) lower than that of previous studies^25^, which ensured we did not underestimate the levels of pesticides in HM. The GC-MS/MS method was also optimized for HM which allows the simultaneous screening of 88 different pesticides within a single injection.

The fat content of HM is the most variable component in HM and is known to be higher in postfeed milk as we have demonstrated^26^. HM *p,p’*-DDE concentrations were also significantly higher in postfeed milk than prefeed milk (Fig. 2A), suggesting an association between lipophilic pesticides (e.g. *p,p’*-DDE) and the fat content of HM. These results support the current hypothesis that POPs are encapsulated either in the core (triacyglycerol) or adhere to the surface (phospholipids) of HM fat during milk synthesis^27^,^28^. Thus after normalization of *p,p’*-DDE to the HM fat content, the significant difference of *p,p’*-DDE concentration between pre- and postfeed milk disappeared (Fig. 2B). Therefore, either pre- or postfeed milk can be collected for the measurement of pesticides provided the concentrations are normalized to the fat content of HM.

We detected large individual variation in HM pesticide concentrations, for example *p,p’*-DDE concentrations ranged from 5.67 to 278.48 ng/mL, and the types of pesticides (e.g. *p,*p’-DDT, *p,*p’-DDE and *β*-HCH), which likely reflect the differences in exposure, lifestyle, dietary habits, travel and metabolic activity between mothers^29^.

As *p,p’*-DDE is a metabolite of *p,p’*-DDT, the 2 mothers with detectable HM *p,p’*-DDT also had higher *p,p’*-DDE concentrations of 125.34 ng/g fat and 80.50 ng/g fat respectively as expected. The ratio between DDE and DDT is commonly used as an indicator of DDT exposure history, where a high DDE/DDT ratio (>5) represents historical exposure and low DDE/DDT ratio (<5) suggests recent exposure^30^,^31^. The DDE/DDT ratio of the two mothers with detected DDT was 3.4 and 8.1 respectively. Both mothers had different exposure characteristics, where one mother frequently visits a country that still has documented high levels of DDT (DDT/DDE ratio of 3.4), while the other grew up on a farm and was involved in the harvesting process during the period from 2004 to 2010 (DDT/DDE ratio of 8.1). These are possible exposure sources of DDT are however based on maternal recollection and further investigation is required including sampling of lactating women living in agricultural regions.

Hexachlorocyclohexane (HCH), is widely used in Australia for insect control^18^. Among its isomers (*α-*HCH, *β*-HCH, *γ-*HCH and *δ-*HCH), *β*-HCH is the most metabolic stable and persistent, and accounts for over 90% of the total HCH detected in HM^32^,^33^. Similar to DDE/DDT exposure history, the *β*-HCH/*α*-HCH ratio is used as an indicator of HCH exposure history^34^. Since *α*-HCH and *γ*-HCH were not detected in this study, suggesting that the mother in whom we detected *β-*HCH had historical exposure to HCH (*β*-HCH/*α*-HCH ratio: >5).

Over the first 12 months of lactation, there was an overall trend for the detected POPs in HM to steadily decline for *p,p’*-DDE (68%), *p,p’*-DDT (45%) and *β*-HCH (73%) suggesting a reduction in maternal body burden^35^,^36^. However, we only observed significant differences between the time points 2 and 12 months for HM *p,p’*-DDE concentrations. This may be attribute to the fact that 10 women showed a decline in HM POPs, while the other 6 showed fluctuations possibly due to change in diet or metabolic activity. Increasing the sample size may increase the ability to detect differences between the months. These results are consistent with previous studies that have shown reductions of pesticides concentration in HM over shorter periods of time such as a decrease in *p,p’*-DDE and *p,p’*-DDT in colostrum (day 4/5) to week 2 by 4% and 10% respectively^37^. Similarly a substantial decrease of *p,p’*-DDE between colostrum (day 3) and week 3 of 22% has been documented^38^,^39^,^40^,^41^,^42^. While extensive studies have been carried out on dioxins and furans^43^, PCBs^44^,^45^ and polybrominated diphenyl ethers (PBDEs)^44^. There are limited studies that have investigated pesticide levels in HM which extend to a period of more than 6 months of lactation and have a sufficient number of participants^45^,^46^. This is one of the more extensive studies which measured pesticides in HM from 16 mothers, longitudinally over 12 months, providing information relevant to the longer term development of the infant in accordance to the WHO recommendation of breastfeeding for up to 1 years and beyond^47^.

Many factors, such as maternal age, parity and BMI, have been associated with HM pesticide concentrations. Previous studies have reported associations between HM *p,p’*-DDE and maternal age^48^,^48^, suggesting increased bioaccumulation of pesticides with increasing age. Further, increased maternal parity has been associated with lower HM pesticides concentration, which is attributed to the increased excretion of body stores through multiple pregnancies and lactations^22^,^42^, whereas increased BMI has been associated with lower HM pesticides concentration. In this study we found no association between HM *p,p’*-DDE and maternal age (*P* = 0.06) or parity (*P* = 0.65) although maternal age is borderline. The absence of a relationship between maternal age and HM POPs may be due to the small number of participants (n = 16) in this study. As body composition measurements, such as BIS are better measures of fat mass we hypothesized that we might find a relationship between fat mass and HM pesticides. However, we were unable to find a relationship between HM *p,p’*-DDE and maternal BMI (*P* = 0.27) and maternal fat mass (*P* = 0.08). These results are similar to that observed by Dirinck *et al*. where pesticide levels in maternal serum were investigated with respect to BMI and fat mass^50^. A greater sample size may determine whether or not maternal adiposity is associated with HM pesticides content.

Many studies have reported the adverse effect of pesticides by associating prenatal exposures with infant growth and development^39^,^51^,^52^. However, none has evaluated the potential influence of these pesticides in HM on the infant growth throughout the first year of lactation. In this cohort, we saw significant increases in infant growth outcomes, such as weight, length and head circumference, over the first 12 months as expected. However, none of these measures were associated with HM levels of *p,p*’-DDE. Further utilizing 2 different measures of infant adiposity (ultrasound skinfolds and BIS), we found no relationship with HM *p,p*’-DDE. These findings may be influenced by the fact that the levels of pesticides detected in HM in this study were at trace levels and the small numbers of participants (n = 16). Whilst associative relationships do not indicate causation, it is reassuring that the low levels of pesticides detected in WA women’s milk do not appear to be of concern.

Whilst the levels of the detected pesticides in HM in this study were very low, HM is the sole food source for a breastfed infant in the first 6 months of life and contributes substantially to the infant diet in the following 6 months^53^, thus it is important to consider the dose of ingested pesticides. To get a more accurate measure of the dose, we measured both the fat content of the milk and the 24 hour intake of the infant at 2 to 5 months, where intake has been shown not to change during this time period^40^, and 9 and 12 months and substitute the estimated values with actual values in the formula for estimated daily intake^41^,^42^. This provides a more accurate calculation of the actual daily intake (ADI) by the infant as it is well documented that both the fat content of milk and the daily volume of milk vary 3-fold between breastfeeding infants^54^. A more accurate quantification for the daily intake of pesticides in infant is essential as studies assessing the effects of pesticides in HM are currently inaccurate and have the potential of not detecting an effect or producing an effect that is not real. Further effects of pesticide must be carefully monitored before implementing interventions to reduce both maternal and infant exposure. As expected, we found a significant difference between ADI and EDI. The EDI, which is based on constant values, underestimated the pesticide intake by 10% at 2 months and overestimated intake by 50 to 233% in the later months (Table 3). The differences are largely due to the overestimation of milk intake at 9 and 12 months of lactation (9 months: 482 ± 76 mL; 12 months: 256 ± 81 mL) for the EDI where a constant of 700 mL is used. Interestingly, the ADI decreased significantly from 2 to 12 months of lactation (Fig. 4), but EDI did not, clearly demonstrating that EDI is not an accurate measure of infant dose. The ADI for several infants exceeded the TDI^55^,^56^ (0.5 μg/kg body wt./day) at 2 and 5 months. However, as the breastfeeding progresses, the ADI at 9 and 12 months are 2 to 25 times lower than the TDI, suggesting that maternal HM pesticides in this cohort poses minimal risk to individual infants in WA. Whilst concern about the effects of pesticide exposure to infants is warranted as one must take into consideration the relatively short period of exposure via breastfeeding relative to a lifetime. Further, HM serves as an important environmental indicator of population exposure.

In order to understand the current magnitude of POPs in HM in WA, the results obtained in this study were compared with those reported in Australia as well as those reported from other countries. The total DDTs in this study have decreased by 93% and 80% as compared to previous studies conducted in WA (1990 s)^21^ and in Australia (2000 s)^22^ respectively. Our findings are also consistent with the trace pesticides levels found in biofluids (e.g. maternal blood and cord blood) and in tissues (e.g. placenta and abdominal) in WA^18^,^57^, which demonstrate the low presence of environmental pesticides and the continuing decline of human exposure to these pesticides over time in WA. As compared to other countries, the concentrations of DDTs observed in this study are similar to those reported for Norway^32^ and USA^58^, but are a few orders of magnitude lower than that observed in malaria-prone countries such as Vietnam^49^, Malaysia^59^ and India^42^ where DDTs was widely used to combat mosquito borne malaria and have only recently been banned in the 1990 s and 2000 s (Table 4). WHO however, have recently allowed limited use of DDT for indoor control of malaria vectors in malaria endemic countries^60^. Therefore, concentrations of DDTs in South Africa^61^ and Ethiopia^62^ are 122 and 330 times higher than that in WA. Concurrent with these DDTs findings, the HCHs concentrations measured in this study are 4 to 63 times lower than the concentrations recorded in countries such as India^42^, Malaysia^59^ and Iran^63^, where HCHs are still extensively used and are commensurate with levels observed in Vietnam^49^, Slovakia^37^ and USA^61^.

The results regarding the levels of pesticides in HM from women in WA, Australia are encouraging however development of analytical methods should continue to focus on reducing detection limits thereby making associative studies more meaningful.

## Conclusions

In this study, out of the 88 targeted pesticides, we have detected trace amounts of *p,p’*-DDE, *p,p’*-DDT and *β*-HCH in HM collected from Western Australian women, and the levels of these pesticides decreased substantially throughout the first year of lactation. This is the first longitudinal study to investigate the relationships of detected pesticides on the infant growth outcomes such as weight, length, head circumference and body composition of which we found none. Further, EDI dramatically overestimates infant dose after 2 months of lactation while the more accurate ADI show reduced dose to infants. The ADI of pesticides for individual infants in Western Australia decreased significantly during the first year of lactation, and was 2 to 17 times lower than current recommended guidelines

## Materials and Methods

### Chemicals and reagents

The pesticide standard solutions (100 μg/mL) at 95% or higher purity were obtained from Ultra Scientific (North Kingstown, RI, USA). Pesticide standard solutions (100 μg/mL) were mixed and diluted with acetonitrile (ACN) to prepare a stock standard solution (1 μg/mL) of all the pesticides. LC-MS grade acetonitrile (ACN) and water were purchased from Thermo Fisher Scientific (Waltham, MA, USA). Ethylglycerol (98%), gulonolactone (95%) and D-sorbitol (≥98%) were from Sigma-Aldrich (St. Louis, MO, USA). Sodium acetate (>99.0%) and magnesium sulfate (99.5%) were purchased from Sigma-Aldrich (St. Louis, MO, USA). Octadecylsiyl (C18) and primary secondary amine (PSA) were obtained from Agilent (Little Falls, DE, USA). Isotopic labeled quality control (QC) standards, acenaphthene-D_10_, phenathrene-D_10_ and chrysene-D_12_ were purchased from Restek (Bellefonte, PA, USA), and the internal standard (IS), triphenylphosphate (TPP) was purchased from Sigma-Aldrich (St. Louis, MO, USA).

### Ethics, sample collection and processing

This study was approved by the Human Research Ethics Committee of The University of Western Australia, and the methods were carried out in accordance with the approved guidelines. Western Australian breastfeeding mothers (n = 16) were recruited between 2013 and 2015. All participants provided informed consent and completed a questionnaire including relevant demographic data. Milk samples were collected (1–5 mL) at 2, 5, 9 and 12 months of lactation into glass containers before and after feeding from each breast. HM fat content was measured immediately using the Creamatocrit method^64^, and the remaining milk was stored at −20 °C.

HM samples (n = 99) were thawed at room temperature for 3 hours then homogenized with a mixer (ELMI Ltd., Riga, Latvia) for 15 seconds. 1 mL of HM was put into a 15 mL centrifuge tube and 1 mL of ACN containing 1% acetic acid and 100 ng/mL QC standards (acenaphthene-D_10_, phenathrene-D_10_ and chrysene-D_12_) was added, and the tube was vortexed for 1 min. A validated method based on acetate buffered QuEChERS was employed to extract the HM^24^,^65^,^66^. Extraction reagents (0.4 g MgSO_4_ and 0.1 g NaAc) were added to the mixture and shaken immediately. The tube was then placed in an ice bath to prevent thermal degradation of some pesticides during the salting out process. The extraction tube was centrifuged at 3993 *g* for 10 min. 0.6 mL of the supernatant was transferred into a clean 15 mL centrifuge tube and stored in a freezer (− 20 °C) for 2 hours. The supernatant was centrifuge at 3993 *g* (0 °C) for 10 min and 0.5 mL was transferred to a cleanup tube (157 mg MgSO_4_, 9 mg C18 and 9 mg PSA). The tube was vortexed for 1 min and centrifuged at 3993 *g* for 10 min. The final extract was transferred into a screw cap amber vial and kept at −80 °C until analysis.

### Maternal and infant anthropometric measurements

All maternal and infant anthropometrics measurements were made at 2, 5, 9 and 12 months at the time of milk sampling. Maternal body weight was measured by an electronic scale (Seca, California, USA, accuracy 0.1 kg). The height, age and parity were self-reported by participants. Maternal BMI was calculated as: BMI = kg/(m^2^).

Infant weight was determined by weighing before breastfeeding using electronic scales (±2.0 g; Medela Electronic Baby Weigh Scales, Medela AG, Switzerland). Infant crown-heel length was measured, to the nearest 0.1 cm, on a hard surface with between a head and footpiece with non-stretch tape. Infant head circumference was measured with non-stretch tape.

### Maternal and infant body composition measurements

Whole body bioimpedance (wrist to ankle) was measured with Impedimed SFB7 bioelectrical impedance analyzer (ImpediMed, Brisbane, Queensland, Australia) according to the manufacturer’s instructions however the infants’ whole bioimpedance were analyzed with settings customized for each infant according to Lingwood *et al*.^67^. Resistance at 50 kHz was used in infant percentage fat mass equations^67^,^68^.

Infants’ ultrasound skinfold measurements (triceps and subscapular) were made using Aplio XG (Toshiba, Japan) machine, PLT-1204BX 14–8 MHz transducer and Parker ultrasonic gel (Fairfield, NJ, USA). The double skinfold thickness, measured directly from images using the on screen electronic calipers, was used in percentage fat mass equations developed for skinfolds measured with skinfold calipers^69^.

### 24–hour infant milk intake

24–hour milk intake was determined by the testing weighing procedure as previously documented^70^. Briefly mothers weighed their infants before and after each feed from each breast for a period of 24 hours. The difference in weight in grams is considered equivalent to mL (density of milk: 1.03 g/mL). If the data was unavailable, the data from previous 24–hour milk intake study were used^71^.

### GC-MS/MS analysis and Quality control

Chromatographic separation and determination of the pesticides were carried on a Bruker Daltonics 450 gas chromatography (GC) with a Bruker Daltonics Scion TQ triple quadrupole mass spectrometer (MS), a Bruker 1177 Split/Splitless injector (Billerica, MA, USA) and a PAL Combi autosampler (CTC Analytics AG, Switzerland). Sky 4.0 ID precision inlet liner with wool from Restek (Bellefonte, PA, USA) was used. For the GC separation, a Rtx-5MS with Integra-Guard column (10 m + 30 m × 0.25 mm × 0.25 μm) (Bellefonte, PA, USA) was used. The inlet temperature was kept constant at 250 °C. The initial oven temperature of the column was 80 °C for 3 min and ramped at 30 °C/min to 150 °C and then ramped at 10 °C/min to 300 °C, where it was held for 10 min. The total run time was 31 min. Helium (6.0 GC grade) was used as carrier gas at a constant flow of 1.1 mL/min. The injection was performed at pulsed splitless mode (head pressure; 44 psi) with an injection volume of 2 μL. The mass spectrometer was operated in electron impact (EI) mode. Transfer line and ion source temperature were 270 °C and 200 °C, respectively. Argon was used as the collision gas. Identification and quantification of 88 pesticides were carried out by tandem MS using scheduled multiple reaction monitoring (MRM) mode. The optimized MRM transitions and collision energies for each compound are listed in Supplementary Table 1. Data collection and processing were performance using Bruker MSWS 8 Software.

Working standard solutions (0.5, 1, 2, 5, 10, 20, 50 and 100 ng/mL) were prepared by appropriate dilution of the stock standard solution (1 μg/mL) with ACN. A combination of APs mixture (ethylglycerol, gulonolactone and D-sorbitol) containing internal standard TPP (IS) was added to the final extract of each HM sample and all the working standard solutions for GC-MS/MS analysis. The final concentrations of ethylglycerol, gulonolactone and D-sorbitol were 20, 2 and 2 mg/mL, respectively and the IS was 100 ng/mL. Isotopically labeled quality control (QC) standards were employed to evaluate the efficiency of the extraction and cleanup steps, while TPP (IS) was used to evaluate the performance of the instrument throughout the entire analytical procedure. In the absence of a true “blank” HM matrix, the use of APs mixture was investigated to counteract the matrix effect in HM. Similar peak response were observed between the pesticides spiked (10–100 μg/L) in APs mixture and in HM samples with APs mixture. A QC mixture containing pesticide standards was analyzed between sample batches to check for any interferences and cross-contaminations. The recoveries of majority (88.6%) of the 88 pesticides were within the range of 70–120%. Limit of detection (LOD) and limit of quantitation (LOQ) were assessed experimentally with the lowest standards spiked in APs mixture providing signal-to-noise ratio (S/N ratio) greater than 3 and 10, respectively. The LOD and LOQ of the 88 pesticides spiked in APs mixture were 0.2–2.0 ng/mL and 0.5–5.0 ng/mL respectively.

### Infant daily intake

The ADI (μg/kg body wt./day) of the pesticides for each infant was calculated based on the pesticides concentration in HM (μg/g fat; ***C***_pesticide_), fat content in HM (g/mL; ***C***_fat_), average daily consumption of HM (mL; ***V***_milk_) and body weight of infant (kg; ***M***_infant_) using the following equation:


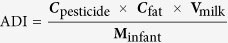


The estimated daily intake (EDI) is based on the same formula as ADI, but was calculated using constant values for ***C***_fat_ (0.03 g/mL), ***M***_infant_ (5 kg) and ***V***_milk_ (700 mL) based on previous studies^41^,^42^.

### Statistical analyses

Statistical analyses were carried out using SPSS software (SPSS, version 19.0 for windows, SPSS, Inc, IL, USA) and R 3.2.0 using the package nlme for linear mixed models which account for intra- and inter-individual variation^72^. Results were expressed as mean ± SD unless stated otherwise. Pesticides that were below the LOD were considered as absent and were not included in the calculations. Detected pesticides were reported based on the HM fat (ng/g fat). Linear mixed models were used to investigate associations between HM pesticide concentrations and both maternal and infant anthropometrics. One-way ANOVA and Tukey’s all pair comparison tests^73^ were used to compare differences in pesticides concentration, EDI and ADI at the different lactation months. Paired samples t-test was used to compare the daily intake of the detected pesticides using the EDI and ADI. *P* < 0.05 was considered significant.

## Additional Information

**How to cite this article**: Du, J. *et al*. Longitudinal study of pesticide residue levels in human milk from Western Australia during 12 months of lactation: Exposure assessment for infants. *Sci. Rep.* **6**, 38355; doi: 10.1038/srep38355 (2016).

**Publisher's note:** Springer Nature remains neutral with regard to jurisdictional claims in published maps and institutional affiliations.

## Supplementary Material

Supplementary Information

## Figures and Tables

**Table 1:** Participant characteristics of a longitudinal study measuring the concentration of pesticides in human milk collected from women (n = 16) at 2, 5, 9 and 12 months postpartum in Western Australia.

	Mean ± SD (Range)			
Mother (n = 16)				
Age	33.9 ± 5.1 (24–43)			
Parity	2.3 ± 1.1 (0–4)			
Infant gestational age (weeks)	39.4 ± 1.4 (38–43)			
Lactation stage (Months)	2	5	9	12
Maternal body mass index (BMI) (kg.m^−2^)	24.9 ± 4.6 (20.4–35.5)	24.3 ± 5.3 (19.0–35.2)	24.5 ± 5.9 (17.9–37.2)	23.7 ± 6.5 (18.2–37.2)
Percentage fat mass (BIS#)	34.4 ± 5.3 (25.7–44.7)	32.8 ± 6.2 (25.1–47.2)	32.6 ± 6.9 (20.0–44.3)	30.2 ± 7.6 (19.4–44.5)
Infant (Male/Female = 9/7)				
Age (Months)	2.0 ± 0.1 (1.9–2.2)	5.1 ± 0.2 (1.8–5.4)	9.2 ± 0.3 (8.8–9.8)	12.2 ± 0.3 (11.6–12.7)
Infant weight (kg)*	5.6 ± 0.9 (4.4–7.4)	7.3 ± 0.8 (6.2–8.7)	8.5 ± 0.9 (6.7–10.1)	9.3 ± 0.7 (8.3–10.6)
Body length (cm)*	57.9 ± 1.8 (54.5–60.0)	64.5 ± 2.4 (60.5–69.5)	70.0 ± 2.3 (66.0–74.0)	73.6 ± 2.4 (69.0–76.3)
Head circumference (cm)*	39.6 ± 1.5 (37.0–42.0)	42.8 ± 1.9 (40.0–45.0)	45.4 ± 1.8 (43.0–48.5)	46.3 ± 1.5 (44.3–49.5)
Percentage fat mass (ultrasound skinfolds)	25.6 ± 4.7 (20.9–37.9)	26.1 ± 3.5 (19.7–33.2)	25.7 ± 4.7 (15.0–34.7)	24.8 ± 4.2 (18.2–30.9)
Percentage fat mass (BIS#)	22.1 ± 2.0 (19.2–24.1)	27.6 ± 2.8 (21.7–30.9)	25.1 ± 5.3 (16.3–33.8)	25.2 ± 3.8 (19.6–30.6)

^#^BIS: bioimpedance spectroscopy.

^*^Significant difference (*P* < 0.05) was observed between months 2–5, 2–9, 2–12, 5–9, 5–12 and 9–12.

**Table 2:** Summary of detected pesticides in HM samples (n = 99) collected from 16 Western Australian mothers.

Pesticide	Frequency of detection (%)	Mean ± SD (ng/mL) (Range)	Mean (ng/g fat) (Range)
*p,p’*-DDE	83	1.56 ± 1.22 (0.21–6.21)	52.25 ± 49.88 (5.67–278.48)
*p,p’*-DDT	9	0.70 ± 0.42 (0.26–1.55)	27.67 ± 20.96 (6.08–69.55)
*β*-HCH	4	2.17 ± 1.69 (0.59–4.55)	48.00 ± 22.46 (14.95–65.08)

**Table 3:** Average calculated daily intake (ADI) and estimated daily intake (EDI) of DDTs by infants during the first year of lactation in comparison to tolerable daily intake (TDI) proposed by FAO/WHO and Health Canada.

Lactation months	ADI (μg/kg body wt./day) (Range)	EDI (μg/kg body wt./day) (Range)	*P* value*	TDI (μg/kg body wt./day) (Range)
All data	0.16 (0.02–0.69)	0.23 (0.03–0.90)	*P* < 0.05	0.5^a,b^10.00^c^20.00^d^
2 months	0.33 (0.07–0.69)	0.30 (0.08–0.90)	0.45	
5 months	0.18 (0.06–0.52)	0.27 (0.08–0.72)	0.08	
9 months	0.09 (0.02–0.22)	0.23 (0.03–0.65)	*P* < 0.05	
12 months	0.03 (0.02–0.05)	0.10 (0.03–0.25)	*P* < 0.05	

^a^US EPA.

^b^RIVM.

^c^FAO/WHO^74^.

^d^Health Canada^41^.

*P value reflects the difference between ADI and EDI, and the difference is significant if *P* < 0.05.

**Table 4:** Comparison of DDTs and HCHs (ng/g fat) in HM from various countries.

Country/region	Year of sampling	Mothers (N)	HM samples	Fat content (g/L)	DDTs	HCHs	References
Iran	2006	23	23	22	2685^a^	3005^A^	53
Indonesia	2003	15	15	na	1100^b^	11^A^	63
Taiwan	2000/01	36	36	31	333^c^	3^B^	24
India	2011	53	53	32	1914^b^	199^B^	48
Vietnam	2000/01	42	42	23	2100^d^	58^C^	36
Malaysia	2003	17	17	17	1600^d^	230^C^	52
Philippine	2004	33	33	22	170^b^	6^A^	23
Slovakia	2003	12	12	27	665^d^	20^C^	34
Norway	2002/06	377	377	36	53^e^	5.4^C^	30
USA	2004	38	38	22	65^a^	19^D^	38
South Africa	2001	28	28	na	6320^c^	12^A^	40
Ethiopia	2010	33	33	na	17170^b^	na	41
New Zealand	2007/2010	39	37	39	385 ^f^	9^A^	64
Australia/WA	2013/15	16	99	39	52^e^	48^C^	Present study

na: data not available.

*Values represented as median value.

^a^Sum of *p,p’*-DDE + *o,p’*-DDE + *p,p’*-DDD + *p,p’*-DDT.

^b^Sum of *p,p’*-DDE + *p,p’*-DDD + *p,p’*-DDT.

^c^Sum of *p,p’*-DDE + *p,p’*-DDD + *p,p’*-DDT + *o,p’*-DDT.

^d^Sum of *p,p’*-DDE + *p,p’*-DDT.

^e^*p,p’*-DDE only.

^f^Sum of *p,p’*-DDE + *o,p’*-DDE + *p,p’*-DDD + *o,p’*-DDD + *p,p’*-DDT + *o,p’*-DDT.

^A^Sum of *α*-HCH + *β*-HCH + *γ*-HCH.

^B^Sum of *β*-HCH + *γ-*HCH.

^C^*β*-HCH only.

^D^Sum of *α*-HCH + *β*-HCH + *γ*-HCH + *δ*-HCH.
