# Supplementary material for: Longitudinal study of pesticide residue levels in human milk from Western Australia during 12 months of lactation: Exposure assessment for infants
Source: Sci Rep. 2016 Dec 7;6:38355. doi: 10.1038/srep38355 (PMC5141434; doi:10.1038/srep38355)
Supplement: Supplementary Information [file srep38355-s1.doc]

**Supplementary material**

**Longitudinal study of pesticide residue levels in human milk from Western Australia during 12 months of lactation: Exposure assessment for infants**

Jian Du ^1^, Zoya Gridneva ^1^, Melvin C.L. Gay ^1, *^, Ching T. Lai^1^, Robert D. Trengove ^2, 3^, Peter E. Hartmann ^1^, Donna T. Geddes ^1, *^

^1^ School of Chemistry and Biochemistry, The University of Western Australia, 35 Stirling Hwy, Perth, Western Australia 6009, Australia

^2^ Separation Science Laboratory, Research and Development, Murdoch University, 90 South St, Murdoch, Western Australia 6150, Australia

^3^ Metabolomics Australia, Western Australia

***Corresponding author:**

Donna Geddes (D.T. Geddes)

Melvin Gay (M.C.L. Gay)

School of Chemistry and Biochemistry, The University of Western Australia, 35 Stirling Hwy, Perth, Western Australia 6009, Australia

**Tel: +61 64887006**

**Fax: +61 64887086**

**E-mail:**

[Donna.geddes@uwa.edu.au](mailto:Donna.geddes@uwa.edu.au)

Melvin.gay@uwa.edu.au

**Table S1**. Retention time and multiple reaction monitoring transitions for 88 pesticides measured in human milk in this cross-sectional study.

| **Group** | **CAS Number** | **Pesticides** | **Retention time (min)** | **Transition ions***  **(Collision Energy, eV)** | **Dwell time (ms)** |
| --- | --- | --- | --- | --- | --- |
| **A** | 319-84-6 | Alpha-HCH | 11.06 | 183.0>147.0 (15)  219.0>183.0 (10) | 53 |
| **A** | 118-74-1 | HCB | 11.21 | 284.0>214.0 (35)  284.0>249.0 (15)  286.0>214.0 (30) | 44 |
| **A** | 319-85-7 | Beta-HCH | 11.57 | 183.0>147.0 (15)  219.0>183.0 (10) | 38 |
| **A** | 58-89-9 | Lindane | 11.70 | 181.0>145.0 (15)  183.0>147.0 (15) 219.0>183.0 (10) | 38 |
| **A** | 76-44-8 | Heptachlor | 13.05 | 272.0>237.0 (20)  274.0>239.0 (15) 274.0>237.0 (5) | 53 |
| **A** | 309-00-2 | Aldrin | 13.73 | 263.0>193.0 (25)  263.0>191.0 (30) 263.0>226.0 (20) | 38 |
| **A** | 1024-57-3 | Heptachlor epoxide B | 14.48 | 353.0>353.0 (5)  353.0>217.0 (20) 353.0>236.0 (10) | 33 |
| **A** | 66240-71-9 | Heptachlor epoxide A | 14.50 | 353.0>353.0 (5)  353.0>217.0 (20) | 33 |
| **A** | 27304-13-8 | Oxychlordane | 14.50 | 187.0>123.0 (20)  387.0>287.0 (15) | 33 |
| **A** | 5103-74-2 | Trans-Chlordane | 14.92 | 373.0>266.0 (20)  375.0>266.0 (20) 373.0>301.0 (10) | 38 |
| **A** | 3424-82-6 | o,p’-DDE | 14.98 | 246.0>176.0 (25)  318.0>246.0 (25) 318.0>176.0 (40) | 38 |
| **A** | 959-98-8 | Alpha-Endosulfan | 15.18 | 239.0>204.0 (15)  241.0>206.0 (10) | 33 |
| **A** | 5103-71-9 | Cis-Chlordane | 15.20 | 373.0>266.0 (20)  373.0>301.0 (10) 375.0>266.0 (20) | 33 |
| **A** | 72-55-9 | p,p'-DDE | 15.54 | 246.0>176.0 (20)  246.0>211.0 (20) | 33 |
| **A** | 60-57-1 | Dieldrin | 15.67 | 263.0>193.0 (30)  277.0>241.0 (10) 277.0>206.0 (20) | 33 |
| **A** | 53-19-0 | o,p'-DDD | 15.73 | 235.0>165.0 (20)  235.0>200.0 (10) | 38 |
| **A** | 72-20-8 | Endrin | 16.10 | 263.0>193.0 (35)  263.0>191.0 (30) 281.0>245.0 (10) | 53 |
|  |  |  |  |  |  |
| **A** | 33213-65-9 | Beta-Endosulfan | 16.27 | 239.0>204.0 (15)  241.0>170.0 (25) 241.0>205.0 (20) | 53 |
| **A** | 72-54-8 | p,p'-DDD | 16.34 | 237.0>165.0 (25)  235.0>165.0 (15) 235.0>199.0 (15) | 53 |
| **A** | 789-02-6 | o,p'-DDT | 16.41 | 235.0>165.0 (20)  235.0>200.0 (10) 235.0>199.0 (15) | 53 |
| **A** | 50-29-3 | p,p'-DDT | 17.03 | 237.0>165.0 (25)  235.0>165.0 (15) 235.0>200.0 (10) | 24 |
| **A** | 115-29-7 | Endosulfan sulphate | 17.04 | 272.0>237.0 (15)  274.0>238.0 (15) 387.0>253.0 (10) | 24 |
| **A** | 72-43-5 | Methoxychlor | 18.05 | 227.0>141.0 (30)  227.0>169.0 (20) 227.0>212.0 (10) | 44 |
| **A** | 2385-85-5 | Mirex | 18.95 | 272.0>237.0 (20)  274.0>237.0 (15) 274.0>239.0 (15) | 133 |
| **B** | 62-73-7 | Dichlorvos | 6.75 | 263.0>191.0 (30)  263.0>226.0 (20) | 266 |
| **B** | 13194-48-4 | Ethoprophos | 10.22 | 158.0>97.0 (18)  158.0>81.0 (15) 158.0>114.0 (10) | 133 |
| **B** | 13071-79-9 | Terbufos | 11.64 | 231.0>129.0 (50)  231.0>97.0 (50) 231.0>79.0 (50) | 38 |
| **B** | 333-41-5 | Diazinon | 11.81 | 304.0>179.0 (10)  304.0>137.0 (35) 304.0>164.0 (35) | 38 |
| **B** | 5598-13-0 | Chlorpyrifos methyl | 12.81 | 286.0>241.0 (25)  286.0>208.0 (10) 286.0>136.0 (20) | 53 |
| **B** | 122-14-5 | Fenitrothion | 13.33 | 260.0>125.0 (15)  260.0>109.0 (10) 260.0>151.0 (20) | 38 |
| **B** | 121-75-5 | Malathion | 13.46 | 173.0>99.0 (18)  173.0>117.0 (10) 173.0>127.0 (10) | 38 |
| **B** | 55-38-9 | Fenthion | 13.68 | 278.0>109.0 (20)  278.0>125.0 (18) 278.0>245.0 (10) | 38 |
| **B** | 2921-88-2 | Chlorpyrifos | 13.71 | 314.0>258.0 (15)  314.0>286.0 (10) 314.0>166.0 (35) | 38 |
| **B** | 34643-46-4 | Prothiofos | 15.36 | 309.0>239.0 (15)  309.0>221.0 (25) 309.0>205.0 (30) | 33 |
| **B** | 41198-08-7 | Profenofos | 15.41 | 339.0>269.0 (10)  339.0>251.0 (25) 339.0>188.0 (20) | 33 |
| **B** | 51-03-6 | Piperonyl butoxide | 17.28 | 176.0>103.0 (25)  176.0>131.0 (10) 176.0>117.0 (18) | 24 |
| **B** | 732-11-6 | Phosmet | 17.91 | 160.0>77.0 (25)  160.0>133.0 (10) 160.0>105.0 (18) | 44 |
| **C** | 2593-15-9 | Etridiazole | 8.54 | 211.0>140.0 (25)  211.0>183.0 (10) 211.0>140.0 (25) | 133 |
| **C** | 23950-58-5 | Propyzamide | 11.73 | 254.0>226.0 (10)  254.0>191.0 (15) 254.0>176.0 (25) | 38 |
| **C** | 1897-45-6 | Chlorothalonil | 12.25 | 266.0>231.0 (18)  266.0>168.0 (25) 266.0>133.0 (30) | 88 |
| **C** | 57837-19-1 | Metalaxyl-M | 13.03 | 206.0>132.0 (20)  206.0>162.0 (10) 206.0>206.0 (5) | 53 |
| **C** | 43121-43-3 | Triadimefon | 13.77 | 208.0>181.0 (10)  208.0>127.0 (15) 208.0>111.0 (25) | 38 |
| **C** | 66246-88-6 | Penconazole | 14.47 | 248.0>157.0 (25)  248.0>192.0 (15) | 33 |
| **C** | 120068-37-3 | Fipronil | 14.50 | 367.0>213.0 (30)  367.0>255.0 (15) 367.0>178.0 (40) | 33 |
| **C** | 55219-65-3 | Triadimenol | 14.56 | 168.0>70.0 (10)  168.0>168.0 (5) | 33 |
| **C** | 76674-21-0 | Flutriafol | 15.19 | 164.0>95.0 (35)  164.0>122.0 (25) | 33 |
| **C** | 79983-71-4 | Hexaconazole | 15.31 | 214.0>172.0 (20)  214.0>152.0 (25) 214.0>124.0 (25) | 33 |
| **C** | 113096-99-4 | Cyproconazole | 15.95 | 222.0>125.0 (20)  222.0>82.0 (10) | 53 |
| **C** | 75881-82-2 | Propiconazole 1 | 16.92 | 259.0>173.0 (20)  259.0>191.0 (10) 259.0>145.0 (40) | 24 |
| **C** | 60207-90-1 | Propiconazole 2 | 17.00 |  |  |
| **C** | 107534-96-3 | Tebuconazole | 17.22 | 250.0>125.0 (15)  250.0>153.0 (10) 250.0>163.0 (10) | 24 |
| **C** | 133855-98-8 | Epoxiconazole | 17.54 | 192.0>138.0 (15)  138.0>111.0 (10) | 29 |
| **C** | 36734-19-7 | Iprodione | 17.72 | 314.0>245.0 (10)  314.0>271.0 (10) 314.0>162.0 (20) | 29 |
| **C** | 95737-68-1 | Pyriproxifen | 18.63 | 226.0>186.0 (18)  226.0>157.0 (30) 226.0>105.0 (10) | 88 |
| **C** | 136426-54-5 | Fluquinconazole | 19.88 | 342.0>300.0 (15)  342.0>315.0 (18) 342.0>288.0 (20) | 53 |
| **C** | 119446-68-3 | Difenoconazole 1 | 22.09 | 323.0>265.0 (15)  323.0>202.0 (30) | 66 |
| **C** | 119446-68-3 | Difenoconazole 2 | 22.19 |  |  |
| **C** | 131860-33-8 | Azoxystrobin | 22.93 | 344.0>329.0 (10)  344.0>156.0 (40) | 133 |
| **D** | 2032-65-7 | Methiocarb 1 | 9.43 | 168.0>153.0 (10)  168.0>109.0 (15) | 133 |
| **D** | 23103-98-2 | Pirimicarb | 12.40 | 166.0>96.0 (15)  166.0>123.0 (10) 166.0>83.0 (18) | 88 |
| **D** | 2032-65-7 | Methiocarb 2 | 13.36 | 168.0>153.0 (10)  168.0>109.0 (15) | 38 |
| **D** | 28434-01-7 | Bioresmethrin | 17.36 | 171.0>143.0 (10)  171.0>128.0 (10) | 24 |
| **D** | 82657-04-3 | Bifenthrin | 17.88 | 181.0>165.0 (18)  181.0>166.0 (10) 181.0>115.0 (40) | 44 |
| **D** | 26002-80-2 | Phenothrin 1 | 18.29 | 183.0>168.0 (15)  183.0>153.0 (150 | 53 |
| **D** |  | Phenothrin 2 | 18.38 |  |  |
| **D** | 52645-53-1 | Permethrin 1 | 19.64 | 183.0>168.0 (10)  183.0>152.0 (20) | 53 |
| **D** |  | Permethrin 2 | 19.76 |  |  |
| **D** | 68359-37-5 | Cyfluthrin 1 | 20.16 | 206.0>151.0 (15)  206.0>179.0 (20) 206.0>177.0 (20) | 38 |
| **D** |  | Cyfluthrin 2 | 20.23 |  |  |
| **D** |  | Cyfluthrin 3 | 20.24 |  |  |
| **D** |  | Cyfluthrin 4 | 20.35 |  |  |
| **D** | 52315-07-8 | Cypermethrin 1 | 20.47 | 181.0>152.0 (20)  181.0>127.0 (30) | 38 |
| **D** |  | Cypermethrin 2 | 20.48 |  |  |
| **D** |  | Cypermethrin 3 | 20.56 |  |  |
| **D** |  | Cypermethrin 4 | 20.66 |  |  |
| **D** | 51630-58-1 | Fevalerate 1 | 21.52 | 225.0>119.0 (18)  225.0>147.0 (10) 225.0>91.0 (25) | 133 |
| **D** |  | Fevalerate 2 | 21.77 |  |  |
| **D** | 173584-44-6 | Indoxacarb | 22.43 | 150.0>123.0 (18)  150.0>150.0 (5) | 66 |
| **D** | 52918-63-5 | Deltamethrin | 22.56 | 253.0>172.0 (10)  253.0>199.0 (25) | 66 |
| **E** | 1582-09-8 | Trifluralin | 10.59 | 306.0>264.0 (10)  306.0>206.0 (15) 306.0>159.0 (30) | 133 |
| **E** | 122-34-9 | Simazine | 11.29 | 201.0>172.0 (15)  201.0>138.0 (10) | 44 |
| **E** | 1912-24-9 | Atrazine | 11.37 | 215.0>200.0 (10)  215.0>172.0 (15) 215.0>138.0 (10) | 44 |
| **E** | 709-97-8 | Propanil | 12.67 | 161.0>99.0 (20)  161.0>126.0 (15) | 66 |
| **E** | 40487-42-1 | Pendimethalin | 14.38 | 252.0>118.0 (30) | 33 |
| **E** | 69806-40-2 | Haloxyfop methyl | 14.75 | 375.0>316.0 (10)  375.0>288.0 (20) | 33 |
| **E** | 79241-46-6 | Fluazifop-p-butyl | 15.83 | 383.0>254.0 (30)  383.0>282.0 (10) | 44 |
| **E** | 51338-27-3 | Diclofop-methyl | 17.22 | 253.0>162.0 (15)  340.0>253.0 (10) | 24 |
| **E** | 83164-33-4 | Diflufenican | 17.23 | 394.0>266.0 (10)  394.0>238.0 (35) | 24 |

A: Organochlorine pesticides (OCPs); B: Organophosphate pesticides (OPPs); C: Fungicides; D: Carbamate pesticides and pyrethroids; E: herbicides and other pesticides.

*: The first transition ion was used for quantification, while the other transition(s) was used as qualifying ion(s).

**Fig. S1.** *p,p’*-DDE (ng/g fat) in human milk between mothers with male and female infant. * Indicates significant difference (*P* < 0.05).

*****

**Fig. S2**. Changes of breastfed infant anthropometric measurements (A) weight, (B) body length and (C) head circumference, from 2 to12 months. ** Indicates significant difference (*P* < 0.01).

******

**A**

******

**B**

******

**C**
